# Differences in rectal fecal microbes among Hu sheep, Tibetan sheep, and their hybrid breeds and their relationship with growth traits

**DOI:** 10.1128/spectrum.01792-24

**Published:** 2025-05-21

**Authors:** Dan Xu, Jiangbo Cheng, Yukun Zhang, Deyin Zhang, Kai Huang, Xiaolong Li, Yuan Zhao, Liming Zhao, Xiaobin Yang, Panpan Cui, Zongwu Ma, Huibin Tian, Xiuxiu Weng, Xiaoxue Zhang, Weimin Wang

**Affiliations:** 1 The State Key Laboratory of Herbage Improvement and Grassland Agro-ecosystems, Lanzhou University, Lanzhou, Gansu, People's Republic of China; 2 Key Laboratory of Grassland Livestock Industry Innovation, Ministry of Agriculture and Rural Affairs, Lanzhou University, Lanzhou, Gansu, People's Republic of China; 3 Engineering Research Center of Grassland Industry, Ministry of Education, Lanzhou University, Lanzhou, Gansu, People’s Republic of China; 4 College of Pastoral Agriculture Science and Technology, Lanzhou University, Lanzhou, Gansu, People’s Republic of China; 5 College of Animal Science and Technology, Gansu Agricultural University 74661, Lanzhou, Gansu, People's Republic of China; Central Texas Veterans Health Care System, Temple, Texas, USA

**Keywords:** hybridization, growth trait, rectal fecal microorganism, 16S rDNA sequencing, host inheritance

## Abstract

Variety and diversity serve as the foundation for animal husbandry development. Nowadays, heterosis is often used to improve animal performance and increase economic benefits. The intestine is an important organ for nutrient absorption and is also the largest immune organ in the body. Intestinal microorganisms can be influenced by various factors such as animal growth and development stages, gender, health status, feed nutrition level, and host genetic background, among others. In this study, three strains of male sheep, with no significant differences in initial weight at 6 months old, were raised under the same conditions until they reached 9 months of age. We collected the rectal feces of these 9-month-old sheep and compared the differences in rectal fecal microorganisms among the three strains using 16S rDNA sequencing technology. The permutational multivariate analysis of variance test revealed significant differences in microorganisms among different strains. Additionally, we identified six biomarkers in Tibetan sheep and Hu sheep, respectively. Functional enrichment analysis showed that microorganisms may affect the growth traits of sheep through lipid metabolism, and the functional differences among different strains were obvious. Therefore, we believe that crossbreeding can result in the offspring obtaining intestinal microorganisms from their parents to varying degrees, and host heredity is the primary cause of the intestinal microorganism differences.

**IMPORTANCE:**

In this study, we identified parental biomarkers by exploring the relationship between parental and hybrid offspring and concluded that these biomarkers may affect related growth traits through fat deposition or lipid metabolism pathways. We also found that hybrid sheep inherited rectal fecal microbes from their parents to varying degrees.

## INTRODUCTION

Along with China’s national economy, the demand for meat products has significantly increased. As a result, high-protein, low-fat, and low-cholesterol lamb is becoming increasingly popular among people. The mutton sheep industry mostly develops through targeted breeding and crossbreeding of local varieties. Therefore, variety serves as the foundation and prerequisite for the development of the meat sheep industry.

Heterosis is the phenomenon in which the heterozygote is superior to the parents in one or more traits (1). In 1876, Darwin first described hybrid advantage after conducting extensive experiments on corn, tomatoes, and other plants with cross-pollination and self-pollination (2). He observed that the hybrid offspring were more advantageous than the self-pollinated offspring. With the rediscovery of Mendel’s law, the study and application of hybrid advantage were further developed. After many scholars’ research and discovery, the hybrid advantage has not only been improved in theory but is also widely used in agricultural production and has become an important way to increase the production of animal husbandry today (3, 4). As early as 1935, there were reports indicating that the production performance of animals was improved, and economic benefits were increased through hybridization to generate heterosis (5). It has also been shown that hybrid offspring tend to exhibit superior growth rates and resistance to stress compared to their parents (6). In a study by Li et al. (7), it was pointed out that introducing genes from Australian Superfine Merino Sheep through hybridization can improve the wool yield and quality of Gansu Alpine Fine Wool Sheep. Additionally, Berganês is produced by crossbreeding Bergamácia sheep and Santa Inês breeds in the semi-arid region of Brazil (8). Kong et al. (9) found that hybrid breeding provided a certain molecular genetic basis for muscle growth and meat quality optimization in sheep. Studies have even found that hybridization can promote global DNA methylation in the skeletal muscle of sheep (10). In addition, hybridization can enhance sheep climate adaptation and resistance to pneumonia (11). This breed has a larger body size and high adaptability to the region. Years of natural and artificial selection have increased the milk production and body size of this hybrid sheep. Therefore, hybridization can alter the production performance of its offspring to varying degrees.

Tibetan sheep is one of the three original sheep breeds in China, mainly distributed in the alpine pasture areas of China, such as the Qinghai-Tibet Plateau (12). It has the advantages of cold resistance, rough feeding resistance, the ability to adapt to a high-altitude environment, and a strong constitution (13). On the other hand, the Hu sheep is a first-class protected local breed in China, with the advantages of early sexual maturity, fast growth, perennial estrus, and strong environmental adaptability (14). It is often used for crossbreeding to improve the lambing rate and produce high-quality lambs due to its multi-lambing nature.

The intestine is responsible for several essential functions such as absorption, metabolism, and immunity (15). Most of the nutrients required by animals need to undergo digestion and absorption in the intestine. After the nutrients from the food are absorbed, the intestine also plays a role in the body’s metabolism by expelling food residues and most toxins through bowel movements. Additionally, the intestinal tract is the largest immune organ in the body. A significant number of immunoglobulins are concentrated in the intestinal mucosa (16), while intestinal microorganisms act as a protective barrier, preventing the invasion of pathogenic microorganisms and maintaining intestinal stability (17). Overall, the intestine performs critical functions that ensure the overall health and well-being of an organism.

The intestinal microbiota is composed of millions of microorganisms that reside in the gastrointestinal tract and continuously interact with the host. These microorganisms, along with their metabolites, play a crucial role in host material metabolism and immune function (18). Intestinal microbes actively participate in the digestion and metabolism of nutrients ingested by the host, breaking down macromolecular substances and producing metabolic products such as short-chain fatty acids, amino acids, and vitamins through their interaction with the host (19–22). Additionally, intestinal microbes act as a barrier, regulating the host’s immune system and influencing the accumulation of specific immune cell groups (23). It is worth noting that the composition and metabolism of the hindgut intestinal flora can often be studied through the analysis of fecal flora since it closely resembles the colon microbiota (24, 25).

This study focuses on Hu sheep, Tibetan sheep, and Tibetan-Hu hybrid sheep as research subjects. By utilizing 16S rDNA sequencing, we aim to investigate the impact of different sheep breeds and breed hybrids on rectal fecal microorganisms and their relationship with economic traits. Additionally, we hypothesize that hybridization can facilitate the acquisition of specific rectal microorganisms from parental breeds, subsequently leading to improved growth and physical characteristics in the offspring. This research holds significant implications for hybrid breeding strategies.

## MATERIALS AND METHODS

### Animals

The two local Chinese breeds of sheep and their hybrids involved in this experiment, including Hu sheep, Tibetan sheep, and Tibetan-Hu hybrid sheep, were selected from Minqin Defu Agricultural Technology Co., Ltd. (Wuwei, Gansu). Rams of similar body weight at 6 months of age were selected from 60 experimental populations. Eight male lambs from each breed, with similar body weight and good physical condition at 6 months of age, were chosen, totaling 24 sheep. All test lambs were immunized through standardized procedures and kept in a single pen of the same size (0.8 × 1 m). The pellet feed eaten during the feeding period was purchased from Gansu Run Mu Bioengineering Co., Ltd. (Gansu, China). The test lambs were fed and watered freely during the feeding period. From 6 to 9 months of age, body weight and body size were measured every 30 days (1 month interval). Measurements were taken on an empty stomach before feeding. Feed was withheld for 12 hours before the measurement to accurately record the amount of food taken by each sheep for each period.

### Sample collection, determination, DNA extraction, and amplification

After the end of the experimental period, disposable PE gloves were used to collect rectal fecal samples from the anus of sheep. After collection, they were temporarily stored in liquid nitrogen (−196°C) and then transferred to −80°C for ultra-low temperature storage. Subsequently, 1 g of rectal feces was weighed, mixed in distilled water in a 1:1 ratio, and shaken well.

The fecal samples were then centrifuged at 5,400 rpm for 10 minutes (radius, *r* = 14.5 cm and relative centrifugal force [RCF] = 4,731 *g*). One milliliter of the supernatant was added with 0.2 mL of a 25% metaphosphate solution containing an internal standard substance (2-ethylbutyric acid). The mixture was mixed well and incubated in ice water for 30 minutes. It was then centrifuged at 10,000 rpm for 10 minutes (centrifuge radius, *r* = 5 cm, RCF = 5,595 *g*). The resulting supernatant was filtered into a 2 mL gas chromatography sample bottle using a 0.45 µm filter membrane. A gas chromatograph (Panna, GC, A91PLUS, China) was used to determine volatile fatty acids in rectal feces. Three milliliters of venous blood from the neck of each sheep was collected and placed in a non-anticoagulant tube. The sample was then centrifuged at 10,000 rpm for 10 minutes to obtain serum, which was analyzed using an automatic biochemical analyzer (Erba, Germany) to measure blood biochemical indicators.

Cetyltrimethylammonium was used to extract DNA from the sample. The concentration and purity of the extracted DNA were analyzed using agarose (GS201-01) gel electrophoresis (*Trans* Marker, BM111-02) and spectrophotometer (Thermo, NANODROP 2000). The sample was diluted to 20 ng/µL with sterile water and stored at −20°C. Using the extracted DNA as a template, the 16S V3-V4 region was amplified using Barcode-specific primers (341F-806R, forward primer F: CCTAYGGRBGCACAG, reverse primer R: GGACTACNNGGTATCTAAT). PCR was performed in a 25 μL reaction volume, consisting of 12.5 μL Phusion High Fidelity PCR-MasterMix (New England Biological Laboratory, Ipswich, MA, USA), 0.4 μL each of upstream and downstream primers, 2 μL template DNA, and 9.7 μL sterile water. The cycling conditions were as follows: denaturation at 98°C for 3 minutes, 21 cycles at 95°C for 30 seconds, 55°C for 30 seconds, and 72°C for 30 seconds,with a final extension at 72°C for 5 minutes. The amplified product was analyzed by 2% agarose gel electrophoresis.

### Sequencing and data processing analysis

Purification and recycling of target bands were performed using the TruSeq DNA PCR-Free Preparation Kit (Illumina, San Diego, CA, USA) to construct a library. The Illumina NovaSeq 6000 sequencing platform was used for high-throughput dual-ended sequencing to generate a paired-end read of 250 bp.

After the original data were taken off the computer, QIIME2 software (version 1.9.1) was used for filtering, and the data were compared with the SILVA database. The UCHIME algorithm was used to remove the detected chimeric sequence, and clean data were obtained for subsequent analysis. Based on the DADA2 algorithm, the effective sequence was denoised to generate amplified subsequence variants (ASVs) with 100% similarity.

Then, the BLAST algorithm was used to compare and annotate the sample sequence with the reference sequence (SILVA138 database). Based on the species annotation situation, QIIME2 software (version 1.9.1) was used for further calculation of α diversity and β diversity. The Bray-Curtis distance was calculated, and RStudio was used for principal coordinate analysis (PCoA) and principal component analysis (PCA). R software (version 4.2.2) was used for data analysis. PCoA and PCA were both performed through the Vegan software package. Linear discriminant analysis (LEfSe) was used to identify biomarkers for each group of sheep. Linear fitting analysis and SourceTracker analysis were performed using R (version 4.2.2). Using PICRUSt2 (version 2.3.0) to predict the function of sample microorganisms based on feature sequences and ASV abundance, this study mainly used the results from the Kyoto Encyclopedia of Genes and Genomes (KEGG) and MetaCyc databases across various fields of life. Multivariate analysis (permutational multivariate analysis of variance, PERMANOVA) was used to analyze the differences between groups. Relevant data have been uploaded to NCBI with the serial number SUB14306888.

Data for blood biochemical indicators and growth traits were analyzed using SPSS 23.0, and significance was compared using the rank-combination test.

## RESULTS

### Effects of hybridization on growth traits and blood biochemical indices of sheep

**Fig 1:**
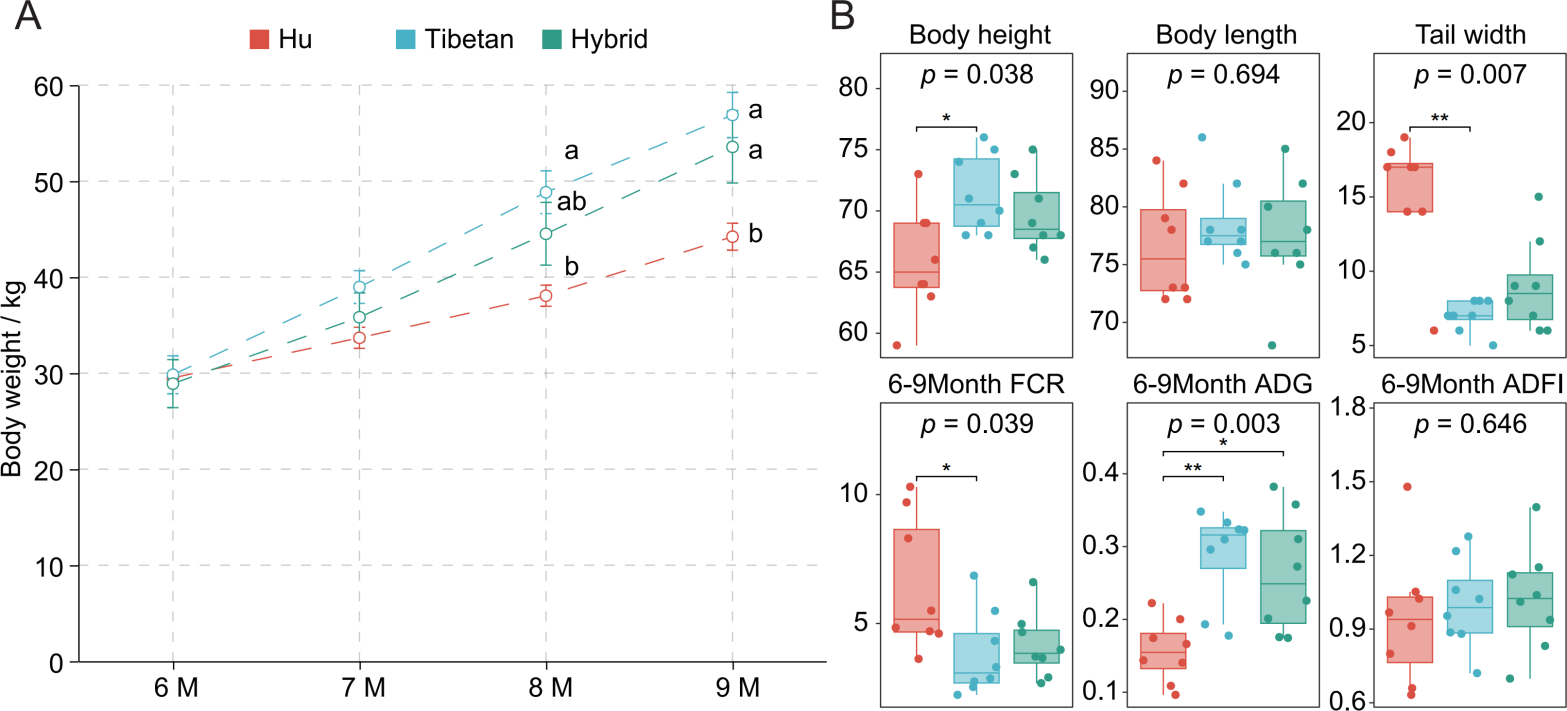
The difference in growth characteristics and blood biochemical indices of different sheep breeds and crossbred sheep. (**A**) Changes in the body weight of sheep aged from 6 to 9 months, different lowercase letters on the same day indicate significant differences among breeds (*P* < 0.05). (**B**) Body size characteristics (body height, body length, and tail width) of different breeds of sheep, and feed efficiency-related characteristics (FCR, ADG, and ADFI) in sheep.

In this cross-sectional study, two parent breeds and hybrid offspring breeds were selected as the research objects. Rams of similar body weight at 6 months of age were selected from 60 experimental populations, and eight sheep were used for each breed, for a total of 24 sheep. We analyzed the trend of body weight of different sheep and the difference in body weight at different stages. The results are shown in Fig. 1A. During the growth stage of sheep from 6 to 9 months, the body weight showed an increasing trend. When the age of 6 months was taken as the starting point of the experiment, there was no significant difference in body weight between the purebred and its hybrid breeds. By 6 months of age, the gastrointestinal microorganisms had colonized and stabilized. At the age of 8 months, there was a significant difference in body weight between Hu sheep and Tibetan sheep (*P* < 0.05), while there was no significant difference between hybrid sheep and the other two breeds (*P* > 0.05). At the age of 9 months, the body weight of Tibetan sheep and hybrid sheep was significantly higher than that of Hu sheep (*P* < 0.05). The body size comparison results showed that the body height of Tibetan sheep was significantly higher than that of Hu sheep (*P* < 0.05), while the tail width was significantly lower than that of Hu sheep (*P* < 0.05), and the hybrid sheep was between the two without significant difference (Fig. 1B) (*P* > 0.05). The feed conversion rate (FCR) of Hu sheep in the experimental stage was significantly higher than that of Tibetan sheep (*P* < 0.05), and the average daily gain (ADG) of Tibetan sheep and hybrid sheep was significantly higher than that of Hu sheep (*P* < 0.05). Interestingly, there was no significant difference in average daily feed intake (ADFI) among the three breeds (*P* > 0.05), indicating that in the experimental stage, Hu sheep had low feed utilization efficiency (Fig. 1B).

In addition, we compared and analyzed the blood biochemical indexes of sheep, including alanine aminotransferase, aspartate aminotransferase, direct bilirubin, total protein, albumin, alkaline phosphatase, creatinine, triglyceride, lactate dehydrogenase, creatine kinase, and blood sugar. The results are shown in Table S1. Among them, the albumin content and triglyceride content of Tibetan sheep were significantly lower than those of lake sheep (*P* < 0.05), and the crossbred sheep was between the other two purebred sheep, with no significant difference (Fig. S1) (*P* > 0.05).

### Overview of rectal fecal volatile fatty acid and microbial sequencing data

The volatile fatty acids in the rectal feces of three breeds of sheep were measured, as shown in Table S2. There was no significant difference (*P* > 0.05) in the volatile fatty acids of each breed.

**Fig 2:**
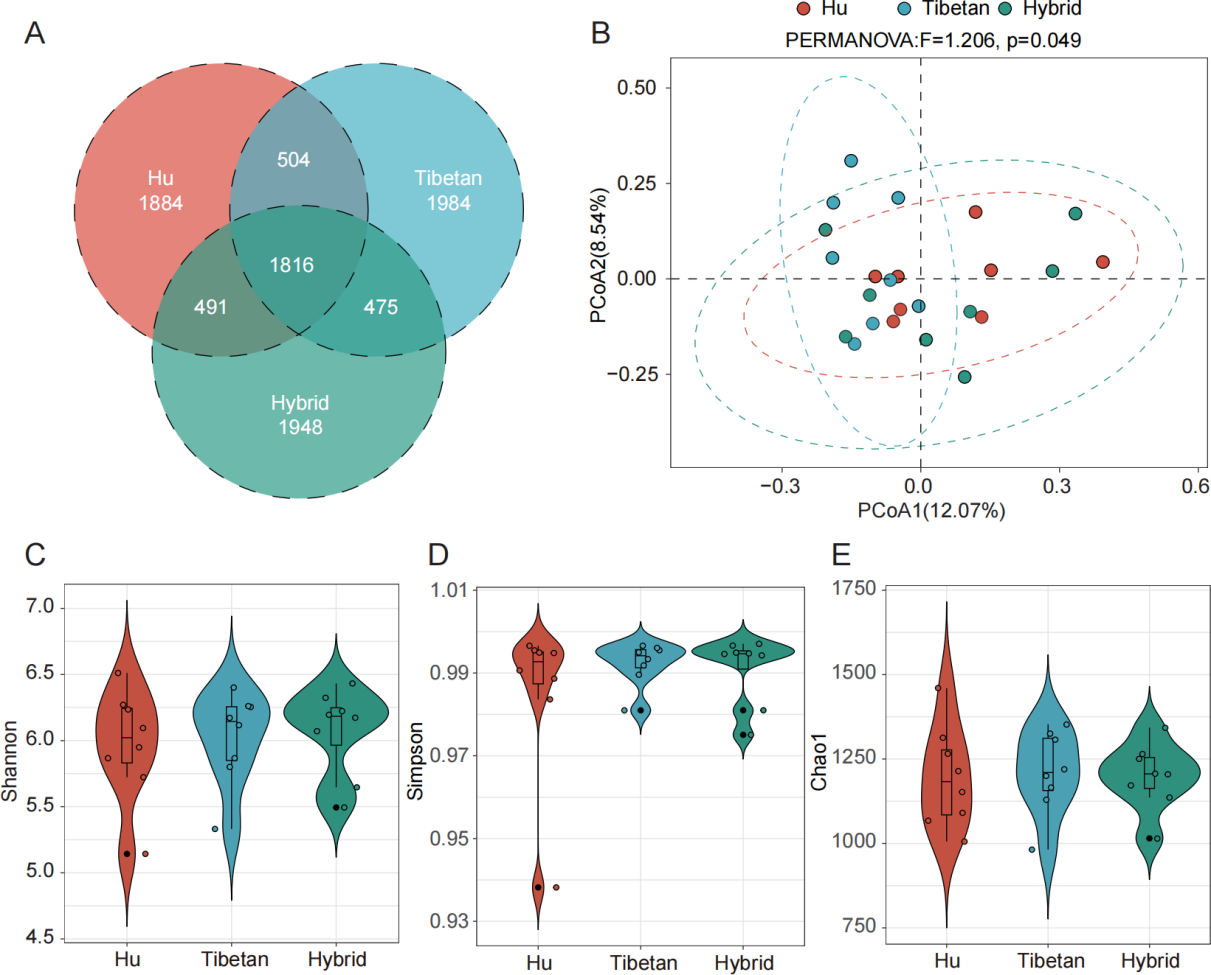
Analysis of the rectal fecal diversity in different sheep breeds. (**A**) Venn diagram of the rectal fecal ASV differences between breeds. (**B**) PCoA plot based on Bray-Curtis distance. The PERMANOVA results are shown at the top. (**C**) Shannon index. (**D**) Simpson index. (**E**) Chao1 index.

The 16S rDNA was amplified based on the rectal feces of three breeds of sheep, and the target was the V3–V4 region of the variable region. High-throughput sequencing was performed on the amplified products, and 1,885,882 raw tags were obtained after splicing. After filtering low-quality sequences and removing chimeras, 1,444,098 effective tags were generated for subsequent analysis. The average sequence length is 413.5 bp (Table S3). After using DADA2 to denoise the sequence, 9,102 ASVs were obtained, among which 1,816 ASVs were common to the three varieties (Fig. 2A). As the sequencing depth increases, the dilution curve tends to be flat, indicating that the sequencing depth can meet the analysis requirements of the samples (Fig. S2).

As shown in Fig. 2B, the separation between Hu sheep and Tibetan sheep was obvious, while the hybrid sheep was between Hu sheep and Tibetan sheep, indicating that the rectal fecal microflora of Hu sheep and Tibetan sheep was different, while the rectal fecal microflora of hybrid sheep was similar to that of the two parent breeds. In order to test the significance of the difference, we used permutational multivariate analysis of variance to analyze the difference between groups, and the results showed that the rectal fecal microflora of the three varieties had significant differences (*P* < 0.05). Alpha diversity index showed that there was no significant difference. Based on the diversity of microorganisms in the samples (*P* ＞ 0.05), the differences in microflora structure among varieties were explored (Fig. 2C through E).

### The rectal fecal microbial composition of purebred and hybrid sheep

**Fig 3:**
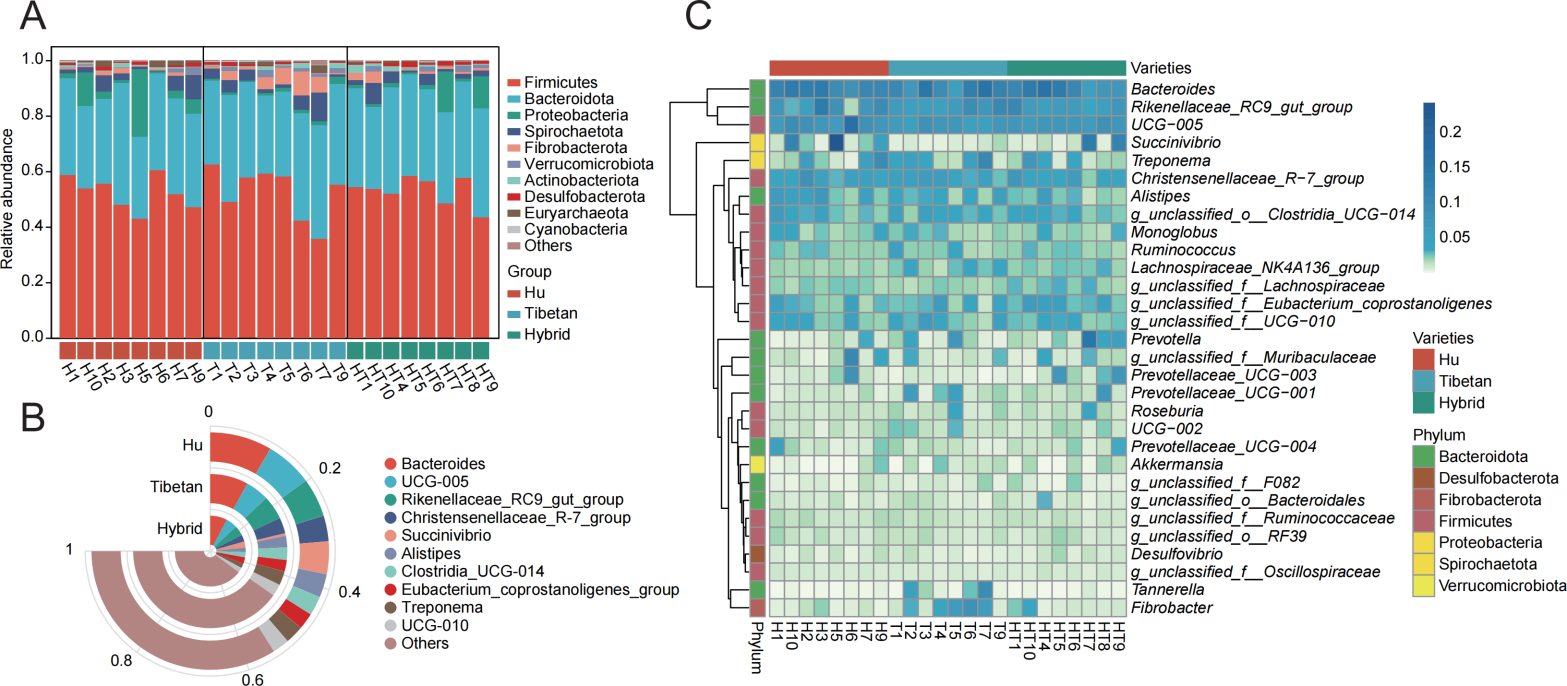
Microbial composition of sheep rectal feces. (**A**) The stacked graph of the relative abundance of the top 10 microorganisms at different phylum levels. (**B**) The stacked graph of the relative abundance of the top 10 microorganisms of different species at the genus level. (**C**) Genus level, top 30 microbial species clustering heat map.

Annotating the obtained effective labels based on the Silva database, a total of 19 phyla, 29 classes, 65 orders, 125 families, 364 genera, and 503 species were obtained. At the phylum level, the dominant phyla of the three varieties were consistent, which were composed of Firmicutes and Bacteroidetes. Proteobacteria had a higher relative abundance in the rectal feces of Hu sheep, while *Fibrobacterota* had a higher relative abundance in the rectal feces of Tibetan sheep (Fig. 3A). At the genus level, the dominant genera of the three sheep breeds were *Bacteroides*, *UCG-005,* and *Rikenellaceae_RC9_gut_group* (Fig. 3B). The relative abundance of the top 30 genera in each sample was normalized, and a relative abundance heat map was drawn. It can be seen from Fig. 3C that the species composition is similar at the genus level among different samples within a variety. The enrichment of *Succinivibrio* in Hu sheep and hybrid sheep was higher, while the enrichment of *Tannerella* and *Fibrobacter* in them was lower. *Prevotellaceae_UCG-001*, *Lachnospiraceae_NK4A136* group, and *Roseburia* were highly enriched in Tibetan sheep and hybrid sheep. This suggests that the microbes in the rectal feces of hybrid sheep may be influenced by the parental sheep breed.

### Effects of hybridization on rectal fecal microbes

**Fig 4:**
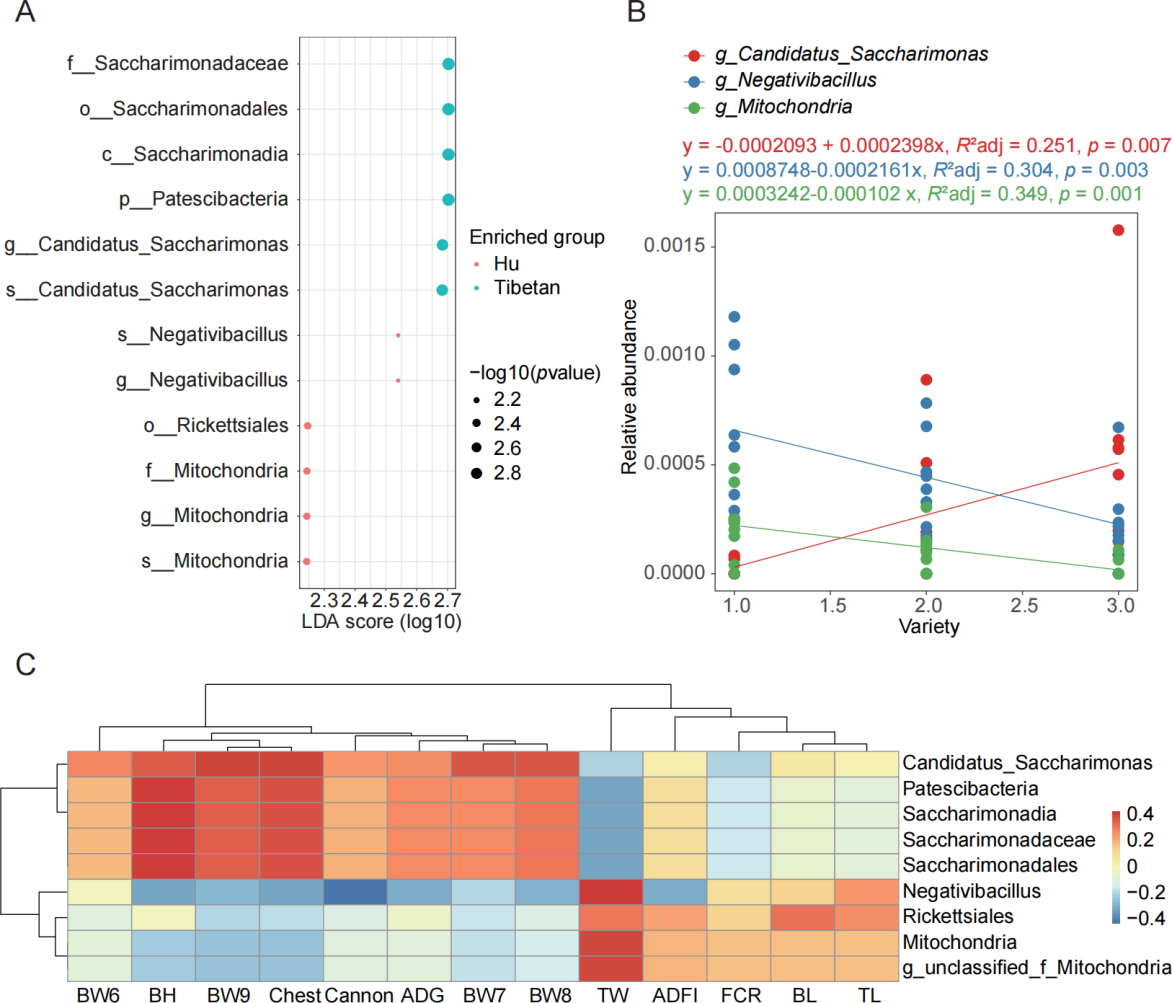
Identification of different breed biomarkers and their relationship to sheep traits. (**A**) LEfSe analysis. (**B**) Linear fit analysis between biomarkers and species. The abscissa indicates the breed: 1, Hu sheep; 2, Hybrid sheep; and 3, Tibetan sheep. (**C**) Correlation analysis between biomarkers and sheep traits.

To explore the relationship between rectal fecal microorganisms between parental breeds and hybrid breeds, we used the LEfSe method to analyze the biomarkers between Hu sheep and Tibetan sheep (LDA score = 2). The results are shown in Fig. 4A. Six biomarkers were identified in the rectal fecal species of Hu sheep and Tibetan sheep, respectively. The biomarkers of Tibetan sheep belong to *Patescibacteria*. The biomarkers identified in Hu sheep at the genus level were *Negativibacillus* and *Mitochondria*. The variety was regarded as an independent variable for linear fitting analysis (Fig. 4B). The results showed that the relative abundance of biomarkers at the genus level showed a significant linear relationship with the cultivars (*P* < 0.05), and the relative abundance of biomarkers in the rectal feces of hybrid cultivars was between the two parental cultivars. Correlation analysis between biomarkers and sheep growth traits was performed, and species-level biomarkers were not used because 16S rDNA sequencing could not accurately analyze species-level microorganisms (Fig. 4C). The results showed that biomarkers such as *Saccharimonadaceae* and *Patescibacteria* in the rectal feces of Tibetan sheep were positively correlated with body weight, body height, chest circumference, daily gain, and other traits, while negatively correlated with tail width and FCR. On the contrary, the biomarker *Rickettsiales* of Hu sheep was positively correlated with tail width and negatively correlated with body weight and other traits. This indicates that hybridization between Hu sheep and Tibetan sheep will change the composition of rectal fecal microorganisms, thereby affecting the growth traits of the host.

### Functional prediction analysis

**Fig 5:**
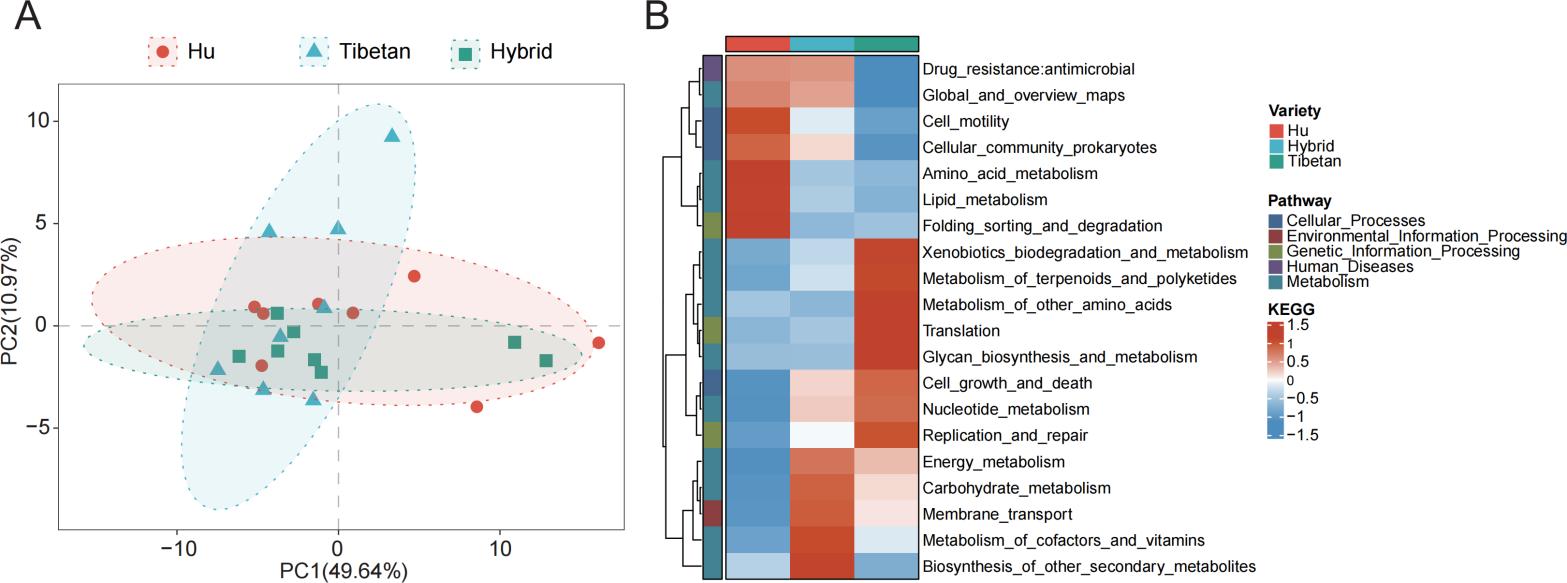
Analysis of differences in functional prediction between groups. (**A**) PCA of microbial functional pathways in different varieties of the MetaCyc database. (**B**) The enrichment heat map of the top 20 functional pathways based on the KEGG database.

Based on the MetaCyc database, we have drawn the PCA map, which shows that the overlapping area between Hybrid sheep and Hu sheep is large, but there are overlapping parts among the three breeds (Fig. 5A). Based on the KEGG database, the microbial functional pathways of different breeds of sheep were predicted (Fig. 5B). In the first pathway, the functional pathway of different breeds of rectal fecal microorganisms was mainly metabolism. The first 20 functional pathways in the second pathway were enriched and analyzed, and the data were unified and standardized, among which amino acid metabolism and lipid metabolism were significantly enriched in Hu sheep. Xenobiotics biodegradation and metabolism, terpenoid and polyketide metabolism, other amino acid metabolism, and Glycan biosynthesis and metabolism are significantly enriched in Tibetan sheep, and energy metabolism, carbohydrate metabolism, cofactors and vitamin metabolism, and other secondary biosynthesis metabolism are significantly enriched in hybrid breeds.

### Correlation analysis between microorganisms

**Fig 6:**
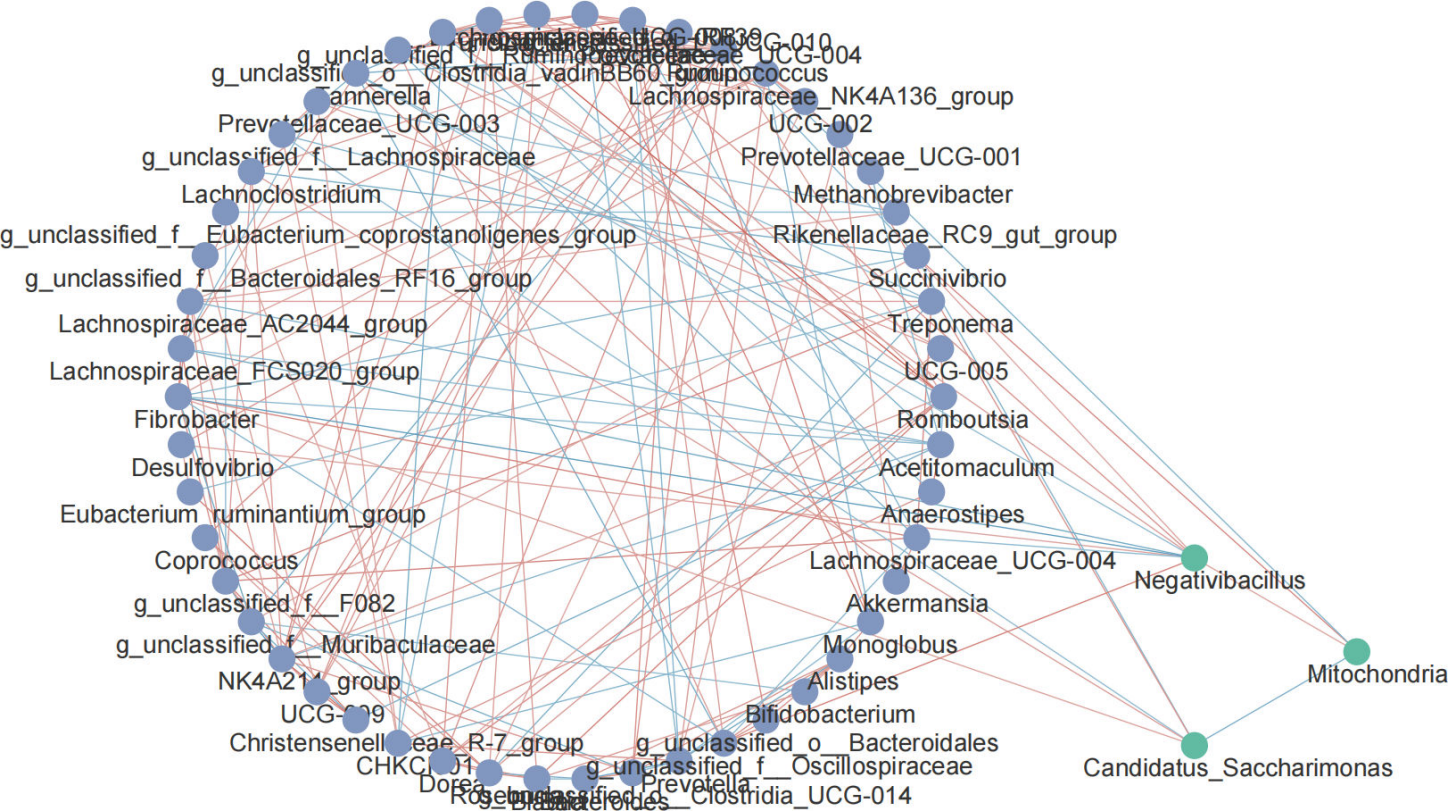
Microbial correlation network diagram at genus level in sheep rectal feces. Purple points are top 50 microorganisms, and green points are biomarkers.

The relationship between biomarkers and rectal fecal microorganisms was explored based on the Spearman correlation coefficient. The top 50 genus-level microorganisms and biomarkers in relative abundance were used to draw a network map. The results are shown in Fig. 6. There is a relatively close correlation between the top 50 microorganisms, and the interaction between different microorganisms constitutes the microbial community in the rectal feces. Among biomarkers, *Mitochondria* was negatively correlated with *Candidatus_Saccharimonas* and positively correlated with *Negativibacillus. Negativibacillus* was significantly correlated with 9 of the top 50 microorganisms (*P* < 0.05), suggesting that it might be a key biomarker affecting the trait.

## DISCUSSION

The results showed that, at 6 months of age, there was no difference in the initial weight of Hu sheep. However, the positive impact of crossbreeding on body weight is evident, suggesting that the use of heterosis breeding can lead to improved performance and greater economic benefits. This is consistent with the research results of Kao et al., which suggest that the hybrid advantage can result in the production of larger-weight hybrid offspring (26). This is advantageous for commercial operations. In addition, the daily weight gain of Tibetan sheep and hybrid sheep is higher than that of Hu sheep. This results in improved feed efficiency in the hybrid offspring. It is evident that as Hu sheep reach a certain stage of growth, their primary focus shifts from growth and development to maintaining daily metabolism and energy intake. On the other hand, Tibetan sheep and hybrid sheep require further development during the later stages of growth. This demonstrates the significant role that effective utilization of heterosis plays in breeding. Additionally, our study observed changes in the tail shape corresponding to the hybridization experiment, indicating that hybridization can directly impact the sheep’s ability to store fat, as evidenced by their physical appearance. In addition, we found that the blood triglyceride levels in Hu sheep were significantly higher than those in Tibetan sheep (*P* < 0.05). The levels of hybrid sheep were lower than those in Hu sheep and higher than those in Tibetan sheep, but there was no significant difference between the hybrid sheep and Tibetan sheep. Triglyceride is one of the most important lipids in the blood, and it is also the most abundant lipid in the body. The fat content in the blood is consistent with the slight trend in tail width, which indirectly suggests that hybridization affects the fat content among different varieties (27). Other studies have shown that regulating the intestinal microorganisms can improve the feed efficiency of pigs (28). The abundance of lactic acid bacteria and *Streptococcus* in the Lagenopsis pigs with different feed efficiency varies (29). Additionally, some differences in flora abundance are also observed in hybrid pigs, which are correlated with feed efficiency (30). Some scholars have pointed out that fat deposition in sheep can be controlled by regulating rumen microbial communities, and certain rumen microbial communities associated with fat deposition have been identified (31). Therefore, we speculate that the difference in gut microbes between parents and hybrids is one of the key factors influencing growth traits.

Studies have shown that various factors, including the growth and development stage of animals (32, 33), gender (34), overall health status, varieties (35, 36), and dietary nutrition level (37), can influence the intestinal microorganisms of animals. These factors can also be influenced by changes in time and space. Cheng et al.’s (25) article analyzing the whole gut microbes of sheep found similar differential biomarkers in the cecum, colon, and rectum, and by 6 months of age, the gastrointestinal microorganisms had colonized and stabilized (38). In order to investigate the potential influence of parental rectal microorganisms on the growth traits of hybrid offspring, we conducted a study to detect and analyze the rectal microorganisms of three different sheep breeds. A PCoA was conducted using the Bray-Curtis distance to assess the diversity and abundance of rectal microorganisms in the three groups. The aim was to investigate the relationship between hybrid breeds and the rectal microorganisms of Hu sheep and Tibetan sheep. A PERMANOVA was used to analyze the differences between the groups. The results showed significant differences in rectal microflora among the three varieties. This finding is consistent with the research results of Cheng et al., who discovered that different varieties have an impact on rumen microorganisms (36). These microorganisms, in turn, affect the growth traits and muscle quality of the host. The results of the diversity analysis showed that there were no significant differences in microbial diversity and richness among the various varieties. This is similar to the research findings of Yang et al. (32). There is no significant difference in the diversity and richness of different species of intestinal microorganisms at the same time, but there is a significant difference over time.

Some scholars have pointed out that at the phylum level, the microbial composition of the pig rectum is similar to that of feces. The dominant phylum is *Firmicutes and Bacteroidetes*, with *Firmicutes* being the most dominant, followed by *Bacteroidetes* (39). This is consistent with our results, and a large number of studies have also supported this view (25, 38, 40). Zhao et al. (39) suggest that *Proteobacteria* is the third most dominant microorganism in adult pig feces, following *Firmicutes* and *Bacteroidetes* (41–43). On the other hand, Niu et al. (44) argue that *Fibrobacterota* is the most dominant microorganism, surpassing *Firmicutes* and *Bacteroidetes*. This discrepancy may be attributed to differences among species. They have detected that the proportion of *Fibrobacterota* in the rectum of Tibetan pigs is significantly higher than that in the other two species. Our study also yielded similar results. Although the species are different, both of them live in high-altitude areas. At the genus level, some studies have shown that *Bacteroidetes* is the dominant flora in the cecum and rectum of small-tailed Han sheep (42). Additionally, some scholars believe that *UCG-005* and *Rikenellaceae_RC9_gut_group* are the dominant flora in the rectum of sheep (38, 45). This is essentially consistent with our research findings.

The experimental results conducted by Wang and his colleagues (46) indicate that the predominant bacteria found in the cecum, colon, and rectum of Qinghai semi-fine wool sheep on the Qinghai-Tibet Plateau are similar. Additionally, *Patescibacteria* is one of the dominant bacteria found in the rectum. It is also noted that a high-fat diet can alter the composition and function of intestinal microorganisms, leading to a decrease in the abundance of *Patescibacteria* in the intestines of mice exposed to such a diet (47). In Tang et al.’s research (48), it was pointed out that high exposure to ammonia disrupts lipid metabolism by activating the mTOR pathway. This leads to an upregulation of genes involved in lipogenesis and a downregulation of genes involved in lipolysis. Additionally, the research found that the number of *Negativibacillus* in the intestine increased.

From our experimental results, it can be inferred that the microorganisms in the rectal feces of hybrid sheep may be influenced by the parental sheep breeds, suggesting a potential role of host heredity. Some scholars replaced 95% of the contents in the rumen of dairy cows and discovered that the similarity of rumen bacterial community composition before and after the exchange of recipient cows was higher than that between donor cows and recipient cows after the exchange (49). Another study found that the estimated heritability of the microbial diversity index, relative abundance, and copy number of total bacteria is greater than 0.15 (35). This suggests that these factors are influenced by the additive inheritance of the host. Therefore, the author concludes that the rumen microbial flora is a heritable factor. In addition to sheep, similar studies have been conducted in other species to investigate the correlation between microorganisms and host genetics (50–53). Researchers believe that specific host genes, such as those associated with the development of the nervous system, immunity, digestion, and fat storage, may serve as the basis for microbial diversity in the gastrointestinal tract of various animals. Macfarlane pointed out in the article that the human intestine is sterile at birth (54). However, it will soon be colonized by microorganisms from maternal feces and the vagina, gradually forming a complex microbial community over time. Tomasello et al. (55) also agree with this view and therefore conclude that this phenomenon is one of the reasons for the significant variations in microbial composition among individuals. The stability of intestinal microorganisms is closely related to the body’s immune function. In experimental research on humans, a correlation has been found between the human microbiota (flora) and genes (56). Related research shows that there are 10 genetic variations in 15 body parts that are significantly associated with microbial composition (57). In the study of siblings, researchers have found that there are groups of bacteria in stool samples that exhibit a high degree of similarity. Furthermore, it has been observed that identical twins tend to have a more similar microbial composition compared to fraternal twins (58). It has been indirectly verified that host genetics play an important role in shaping the gastrointestinal microbial community. Based on the aforementioned perspective, we hypothesize that the genetic makeup of the host plays a crucial role in shaping the composition of the microbial community. To investigate this, we conducted an analysis of biomarkers in Hu sheep and Tibetan sheep. Subsequently, we conducted a linear regression analysis and observed a significant linear relationship between the relative abundance of biomarkers at the genus level and the different sheep breeds. Furthermore, we found that the relative abundance of biomarkers in the rectal feces of hybrid varieties fell between that of the two parent varieties. Therefore, we believe that hybridization has a significant impact on microorganisms, and the hybrid offspring will inherit microorganisms from the parent varieties to varying degrees.

### Conclusion

Our findings revealed differences in the rectal flora among Hu sheep, Tibetan sheep, and their hybrid offspring. We investigated the relationship between rectal flora and growth traits. We observed variations in the rectal fecal microbiota among different sheep strains. Furthermore, we identified six biomarkers in the rectal fecal microbiota of Hu sheep and Tibetan sheep, respectively. The relative abundance of these biomarkers exhibited a significant linear correlation at the genus level between the two parent strains and the hybrid lines. Additionally, these biomarkers may impact sheep’s growth-related traits through fat deposition or lipid metabolism pathways. In summary, hybrid sheep inherit rectal fecal microorganisms to varying degrees from their parents. We believe that this inheritance is closely associated with host heredity and may contribute to the hybrid sheep’s growth traits falling between those of Tibetan sheep and Hu sheep.

## Data Availability

Further data requests and information can be obtained by contacting the corresponding author Weimin Wang at wangweimin@lzu.edu.cn.

## SUPPLEMENTAL MATERIAL

Figure S1 spectrum.01792-24-s0001.tif Blood biochemical indices (total protein, triglycerides) of different breeds of sheep.

Figure S2 spectrum.01792-24-s0002.tif Dilution curve.

Supplemental tables spectrum.01792-24-s0003.docx Tables S1 to S3.

ASM does not own the copyrights to Supplemental Material that may be linked to, or accessed through, an article. The authors have granted ASM a non-exclusive, world-wide license to publish the Supplemental Material files. Please contact the corresponding author directly for reuse.
