# Supplementary figures and images for: Differences in rectal fecal microbes among Hu sheep, Tibetan sheep, and their hybrid breeds and their relationship with growth traits
Source: Microbiol Spectr. 2025 May 21;13(7):e01792-24. doi: 10.1128/spectrum.01792-24 (PMC12210882; doi:10.1128/spectrum.01792-24)

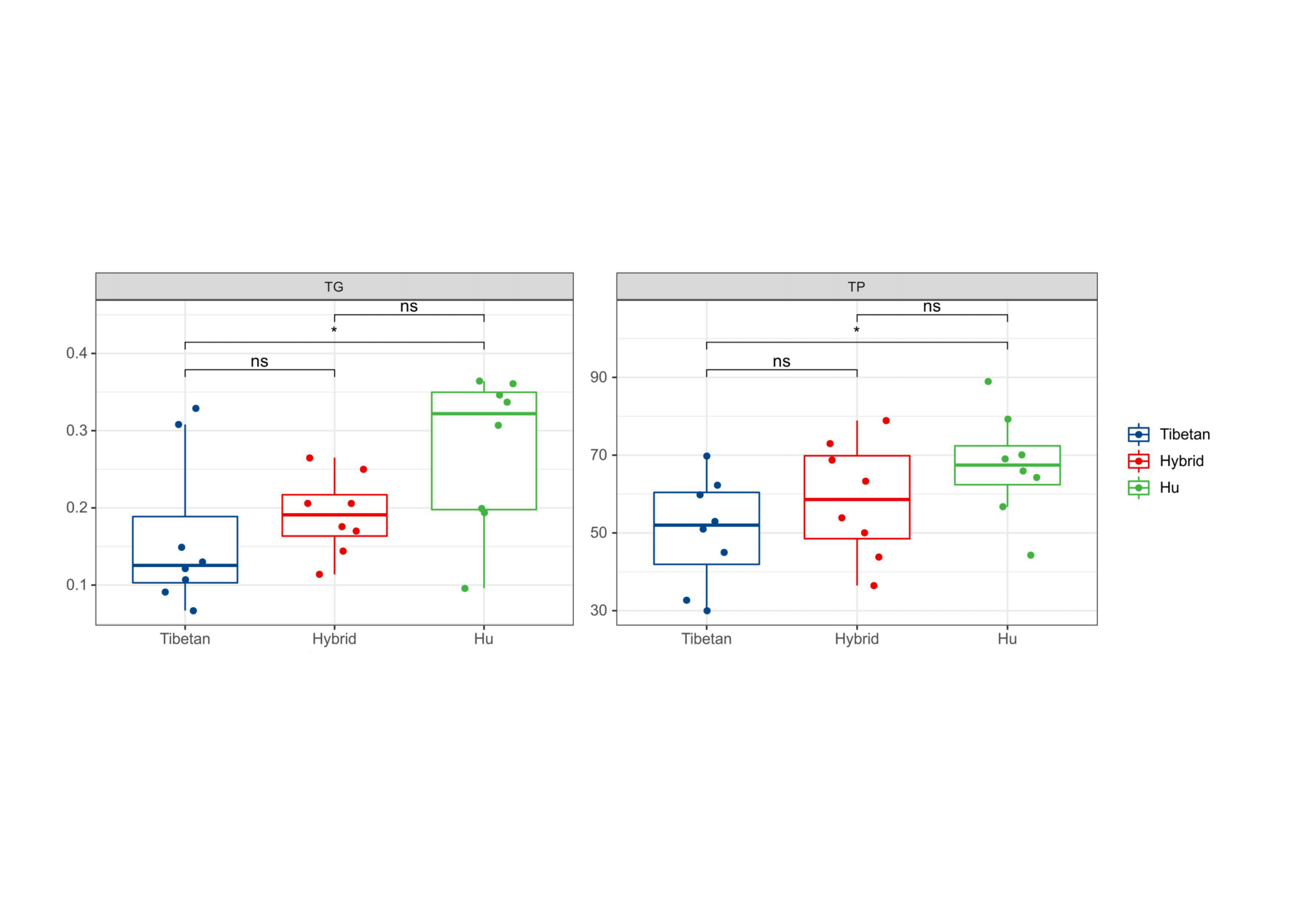

Supplement: Figure S1 — Blood biochemical indices (total protein, triglycerides) of different breeds of sheep. [file spectrum.01792-24-s0001.tif]

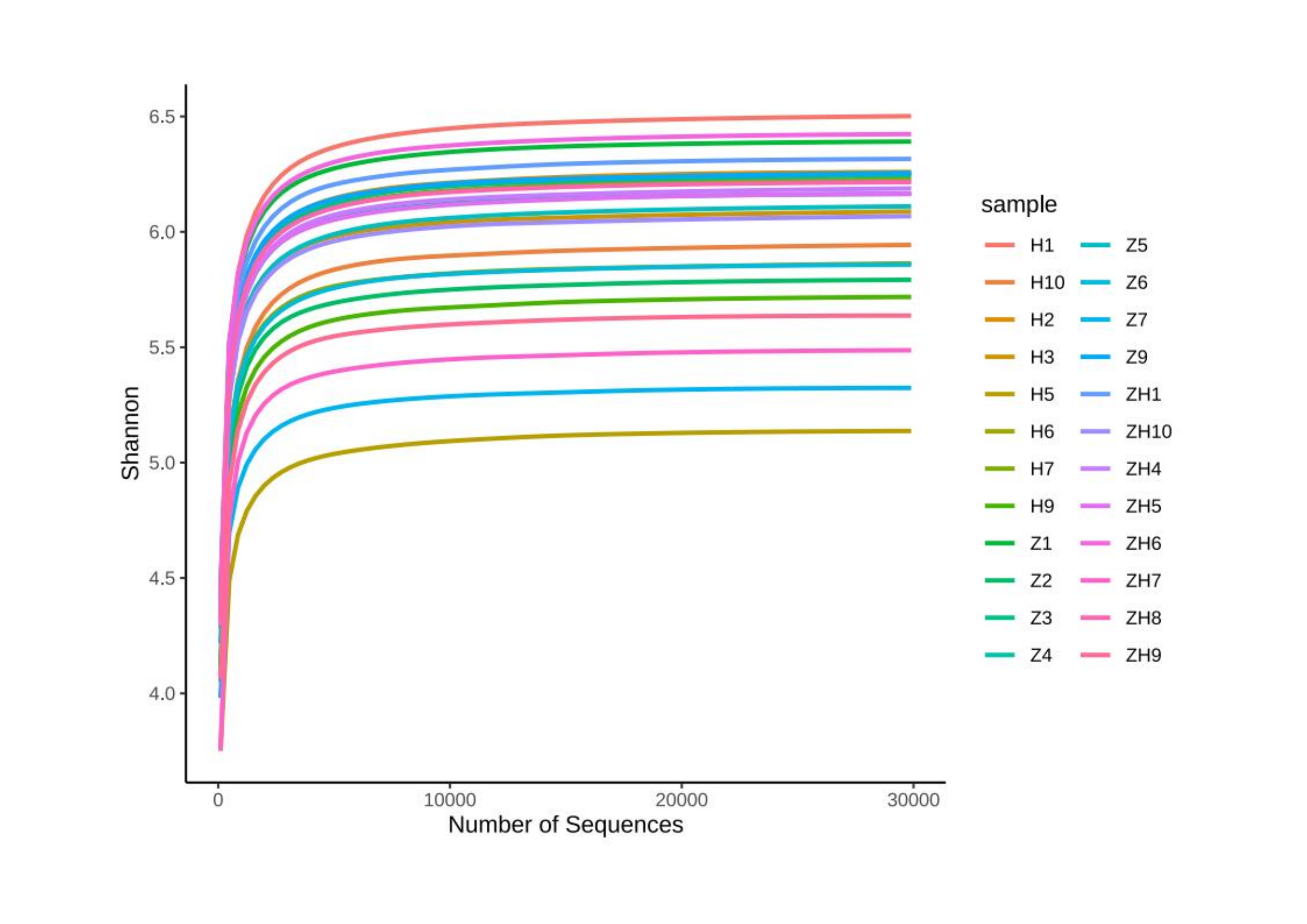

Supplement: Figure S2 — Dilution curve. [file spectrum.01792-24-s0002.tif]
