# Supplementary material for: Differences in rectal fecal microbes among Hu sheep, Tibetan sheep, and their hybrid breeds and their relationship with growth traits
Source: Microbiol Spectr. 2025 May 21;13(7):e01792-24. doi: 10.1128/spectrum.01792-24 (PMC12210882; doi:10.1128/spectrum.01792-24)
Supplement: Supplemental tables — Tables S1 to S3. [file spectrum.01792-24-s0003.docx]

**Supplementary Table 1.** The correlation of blood biochemical indices among different sheep breeds.

| Item | Tibetan sheep | Hu sheep | Hybrid sheep |
| --- | --- | --- | --- |
| ALT,(U/L) | 10.45±5.38 | 10.55±11.98 | 11.55±5.29 |
| AST,(U/L) | 75.78±22.38 | 84.30±27.08 | 88.53±30.73 |
| DBIL,(umol/L) | 1.54±1.11 | 1.25±1.26 | 1.37±0.58 |
| TP,(g/L) | 50.44±13.98^b^ | 67.33±13.51^a^ | 58.54±14.87^ab^ |
| ALB,(g/L) | 20.16±5.30 | 21.03±5.57 | 22.13±4.28 |
| ALP,(U/L) | 261.63±71.55 | 268.38±80.84 | 251.13±75.69 |
| CR,(umol/L) | 35.20±7.92 | 35.43±5.54 | 35.79±4.92 |
| TG,(mmol/L) | 0.16±0.10^b^ | 0.28±0.10^a^ | 0.19±0.05^ab^ |
| LDH,(U/L) | 367.38±89.54 | 378.50±81.51 | 418.63±81.15 |
| CK,(U/L) | 253.61±165.53 | 194.66±77.54 | 266.63±198.07 |
| GLU,(mmol/L) | 3.95±1.27 | 4.07±0.72 | 4.03±0.54 |

*Note*: Measured values are presented as mean ± standard error. In the same row, significant differences are labeled with different lowercase letters (*P<0.05*), or with different capital letters (*P<0.01*).

**Supplementary Table 2.** Rectal fecal fermentation parameters of Tibetan sheep ,Hu sheep and Hybrid sheep.

| Item | Tibetan sheep | Hu sheep | Hybrid sheep |
| --- | --- | --- | --- |
| Acetic acid,% | 60.84±1.92 | 61.46±2.21 | 60.35±3.20 |
| Propionic acid,% | 17.81±1.68 | 16.52±1.31 | 17.04±1.50 |
| Isobutyric acid,% | 1.75±0.42 | 1.78±0.33 | 1.59±0.26 |
| Butyric acid,% | 6.11±1.86 | 7.12±1.11 | 7.05±2.69 |
| Isovaleric acid,% | 1.09±0.17 | 1.27±0.30 | 1.12±0.24 |
| Valeric acid,% | 0.47±0.16 | 0.58±0.14 | 0.54±0.14 |
| TVFA,(mmol/L) | 24.65±3.89 | 26.17±4.28 | 24.48±5.52 |

*Note*: Measured values are presented as mean ± standard error. In the same row, significant differences are labeled with different lowercase letters (*P<0.05*), or with different capital letters (*P<0.01*).

**Supplementary Table 3.** Overview of 16S rDNA sequencing data.

| Sample Name | Raw PE(#) | Raw Tags(#) | Clean Tags(#) | Effective Tags | Base(nt) | Avglen(nt) | Effective% |
| --- | --- | --- | --- | --- | --- | --- | --- |
| Z9 | 82626 | 79768 | 79422 | 60263 | 24906572 | 413 | 72.93 |
| H10 | 80203 | 78563 | 78161 | 56915 | 23465282 | 412 | 70.96 |
| ZH10 | 69184 | 67001 | 66827 | 55121 | 22810305 | 414 | 79.67 |
| Z7 | 82880 | 81708 | 81356 | 58986 | 24535715 | 416 | 71.17 |
| H9 | 76177 | 73915 | 73703 | 60324 | 25020836 | 415 | 79.19 |
| ZH9 | 80270 | 79696 | 79495 | 63742 | 26384445 | 414 | 79.41 |
| H6 | 85423 | 84450 | 84129 | 60737 | 25021899 | 412 | 71.1 |
| ZH8 | 71175 | 68650 | 68454 | 56546 | 23365196 | 413 | 79.45 |
| Z5 | 74408 | 74395 | 74145 | 58864 | 24289036 | 413 | 79.11 |
| H7 | 80792 | 78836 | 78531 | 59940 | 24783418 | 413 | 74.19 |
| Z6 | 78963 | 77032 | 76779 | 61837 | 25697255 | 416 | 78.31 |
| ZH6 | 83612 | 83062 | 82741 | 60543 | 25030244 | 413 | 72.41 |
| Z4 | 82401 | 80523 | 80220 | 62102 | 25595176 | 412 | 75.37 |
| ZH7 | 80107 | 79049 | 78811 | 60976 | 25155530 | 413 | 76.12 |
| Z2 | 82860 | 80182 | 79908 | 60181 | 24983218 | 415 | 72.63 |
| H5 | 70370 | 69749 | 69496 | 51972 | 21387500 | 412 | 73.86 |
| ZH4 | 86664 | 84410 | 84113 | 64016 | 26509503 | 414 | 73.87 |
| Z3 | 84158 | 83127 | 82856 | 61479 | 25397285 | 413 | 73.05 |
| ZH5 | 81581 | 81207 | 80897 | 62028 | 25653486 | 414 | 76.03 |
| Z1 | 78436 | 76748 | 76434 | 58914 | 24316187 | 413 | 75.11 |
| H2 | 83584 | 81722 | 81410 | 62469 | 25811103 | 413 | 74.74 |
| H3 | 80755 | 79237 | 78997 | 63472 | 26351219 | 415 | 78.6 |
| ZH1 | 78116 | 75841 | 75597 | 60002 | 24800033 | 413 | 76.81 |
| H1 | 87772 | 87011 | 86615 | 62669 | 25893247 | 413 | 71.4 |

Figure S1. Blood biochemical indices (total protein, triglycerides) of different breeds of sheep.

Figure S2. Dilution curve.
